# Paper-Based Assessment of the Effects of Aging on Response Time: A Diffusion Model Analysis

**DOI:** 10.3390/jintelligence5020012

**Published:** 2017-04-10

**Authors:** Judith Dirk, Gesa Katharina Kratzsch, John J. Prindle, Ulf Kröhne, Frank Goldhammer, Florian Schmiedek

**Affiliations:** 1 Department of Education and Human Development, German Institute for International Educational Research (DIPF), 60486 Frankfurt am Main, Germany; schmiedek@dipf.de; 2 Department of Psychology, Goethe University, 60232 Frankfurt am Main, Germany; gesa.k.kratzsch@stud.uni-frankfurt.de; 3 Suzanne Dworak-Peck School of Social Work, University of Southern California, Los Angeles, CA 90007, USA; jprindle@usc.edu; 4 Department of Educational Quality and Evaluation, German Institute for International Educational Research (DIPF), 60486 Frankfurt am Main, Germany; kroehne@dipf.de (U.K.); goldhammer@dipf.de (F.G.); 5 Centre for International Student Assessment (ZIB), 60486 Frankfurt am Main, Germany

**Keywords:** processing speed, paper-based assessment, aging, diffusion model

## Abstract

The effects of aging on response time were examined in a paper-based lexical-decision experiment with younger (age 18–36) and older (age 64–75) adults, applying Ratcliff’s diffusion model. Using digital pens allowed the paper-based assessment of response times for single items. Age differences previously reported by Ratcliff and colleagues in computer-based experiments were partly replicated: older adults responded more conservatively than younger adults and showed a slowing of their nondecision components of RT by 53 ms. The rates of evidence accumulation (drift rate) showed no age-related differences. Participants with a higher score in a vocabulary test also had higher drift rates. The experiment demonstrates the possibility to use formal processing models with paper-based tests.

## 1. Introduction

### 1.1. Age-Related Cognitive Slowing and the Diffusion Model

The finding that older adults, on average, perform more slowly than younger adults in speeded cognitive tasks is one of the mostly studied and highly solid phenomena in cognitive aging research [1,2]. Research addressing this phenomenon dates to the late 1960s [3,4] when Birren and others reported evidence that the slowing of behavior in healthy older adults is ubiquitous in all components of processing, ranging from initial encoding to response execution. Since then, different models and theoretical accounts of age-related slowing have been proposed. Cerella’s linear rate model [5] and Salthouse’s processing speed theory of cognitive aging [6] are two of the most prominent accounts. Despite differences in the processes assumed to account for age-related slowing, all theoretical accounts introduce a general slowing factor describing older adults’ performance as an approximately linear function of younger adults’ performance. This general slowing factor has often been illustrated in a so-called Brinley plot in which older adults’ mean reaction times (RTs) in a task condition are plotted on the y-axis, as a function of the corresponding younger adults’ mean RTs on the *x*-axis. The Brinley function is approximately linear with a slope of around 1.5 across a variety of tasks [5]. Given that this pattern has been observed across many different cognitive tasks, cognitive aging researchers postulated a single general mechanism may account for age-related changes in information processing in various cognitive processes (i.e., the generalized slowing hypothesis). However, it is surprising that in this research tradition typically either only response times or the accuracy of responding were considered and different speed-accuracy trade-offs of younger and older adults were largely ignored. As Ratcliff and colleagues argued theoretically [7] and showed empirically (e.g., [8,9,10]), it is indispensable to take a comprehensive approach to RT data including not only the correct RTs but also the accuracy of responses, RTs of incorrect responses, and the shape of the corresponding RT distributions in order to understand the processes underlying the observed cognitive slowing phenomenon. Ratcliff and colleagues demonstrated in several studies and for different experimental paradigms (e.g., [10,11,12]) that the observed cognitive slowing can at least partly be explained by a more conservative response style resulting from a preference for accuracy over speed. In different two-choice RT paradigms, the authors applied the so-called Ratcliff diffusion model [13] (see next paragraph) and found that in many cognitive tasks age differences cannot be localized in the efficiency of the decision process (i.e., the *drift rate* parameter) but result from a more cautious response style (i.e., differences in the *boundary separation* parameter) as well as slower sensorimotor processes involved in the execution of the task (i.e., differences in the *nondecision time* parameter). In sum, they argued, for certain paradigms, it is not the efficiency of the decision process that declines with increasing age. Rather, age-related changes in decision criteria and nondecision components can account for age-related slowing. 

### 1.2. The Diffusion Model

**Figure 1:**
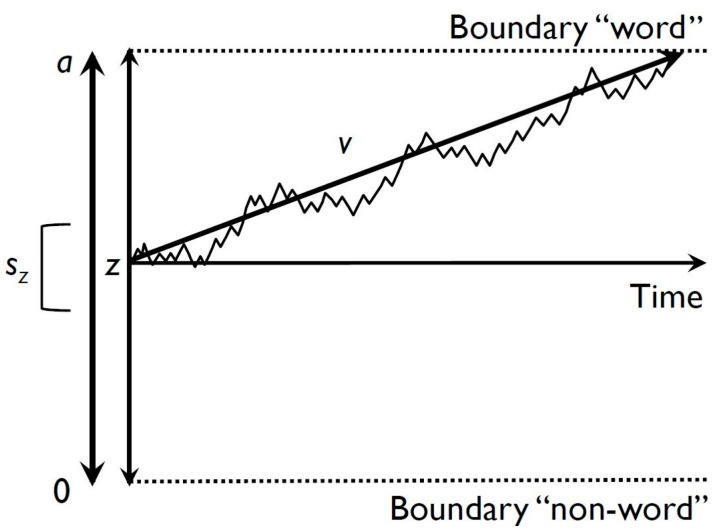
Illustration of the central parameters of the diffusion model for a lexical decision task. Note: *a* = boundary separation, *z* = starting point *s_z_* = variation in starting point, *v* = drift rate. Note that the nondecision time *t*_0_ is not illustrated since it also involves processes of response preparation.

The diffusion model, introduced by Ratcliff [13], describes how the information provided by a stimulus is accumulated over time in the cognitive system until it reaches one of two response boundaries. It takes correct RTs, the accuracy of responses, RTs of incorrect responses, and the shape of the corresponding RT distributions into account to estimate different parameters underlying the cognitive processes involved in two choice RT tasks. Among these parameters, the three most important ones are the drift rate (*v*), the boundary separation (*a*), and the nondecision time (*T_er_*, see Figure 1). The drift rate describes the average rate of accumulation of evidence over time and thus reflects the efficiency of the decision process. Larger drift rates indicate faster accumulation of information and thus higher efficiency. An individual’s response style is reflected by the boundary separation, which is the distance between the two response alternatives (e.g., word vs. nonword in a lexical decision task). Larger boundary separations reflect a more conservative response style and indicate an individual’s need for more information to make a decision. By moving the boundaries, the diffusion model can account for speed-accuracy trade-offs. Associated with the boundary separation is the starting point parameter (*z*), which describes whether there is an a-priori preference (i.e., bias) for one of the two response alternatives. If two response alternatives are equally likely and there is no preference for one over the other, the starting point is in the middle of the two boundaries and can be expressed as *a*/2. When boundaries are further from the starting point, responses are slower errors are less likely. The nondecision time is the third central parameter and reflects sensorimotor processes necessary for sensory encoding as well as motor response preparation and execution. Beyond these central parameters, the model also includes parameters for across-trial and within-trial variability e.g., [14]: variation in the starting point can be described by parameter *s_z_*. This allows us to model variation in the starting point across trials, which can, for example, explain fast error responses [15]. Similarly, across-trial variability in drift rate (*s_v_*) can account for error responses that are slower than correct responses [7]. Within-trial variability in drift rate (*s*) serves as a scaling parameter for the diffusion model parameters and is usually set to 0.01; however, in some modeling software (e.g., *fast*-*dm* [16,17]) it is set to 1. Finally, across-trial variability in nondecision time (*s_t_*) is introduced into the model to explain variation in sensory encoding and response preparation and execution across trials.

### 1.3. Motivation for the Current Study

Most of the above-cited studies on cognitive aging and age-related slowing applied computer-based cognitive testing. Despite the common use of computer-based tasks in cognitive aging research, paper-based cognitive tests of processing speed are still commonly used in cognitive aging research and practice. They are still a standard assessment tool since they are both time and cost efficient ways to assess cognitive performance. Moreover, age-related differences in affinity and use of computers persist and might bias results of studies comparing younger and older adults’ cognitive performance based on computer-based assessment. Paper-based assessment allows eviting such potential bias (*mode effect*, see e.g., [18]). Mode effects for speed tests are more likely than mode effects for power tests [19]. Age-related differences in the mode effect were discussed from the beginning of mode comparability studies (see, e.g., [20]) and were, for instance, found for self-reported measures [21]. As discussed by Fazeli et al. [22] mode effects might be caused by a relationship between computer use and cognitive performance, and age as well as cohort effects are expected in (self-reported) computer use [23]. However, for a long time paper-based assessment did not allow for the assessment of RTs for single items, which is a necessary precondition to apply formal models such as the Ratcliff diffusion model and thereby determine what processes underlie age-related differences in cognitive performance. The assessment of RTs for single items in paper-based tests can now be realized by digital pens using the Anoto technology [24,25,26]. This study used the pen technology to assess age differences in cognitive performance and applied Ratcliff’s diffusion model to the data. To compare the results of the diffusion model to previously reported findings on age-related cognitive performance differences, we chose to apply a lexical decision task for this study. Lexical decision tasks have been previously used with the diffusion model [8,27] and present one of the rare tasks used in the diffusion model and the cognitive aging literature (e.g., [28]) that can be applied in paper-based as well as computer-based testing. Applying the diffusion model to a lexical decision task, Ratcliff, Thapar, and colleagues [8] found older adults to be slower (RT) and more accurate than younger adults, replicating previous findings (e.g., [28]). The diffusion model parameters however demonstrated that the observed slowing of RTs was associated with older adults’ longer nondecision time and the larger boundary separation, whereas the accumulation of evidence reflected in the drift rate did not show age-related differences. Thus, older adults’ longer RTs resulted mainly from their more conservative responding (i.e., the larger boundary separation) and not from deficits in the accumulation of evidence. This illustrates the utility of the diffusion model in explaining sources of age-related differences in cognitive processes. However, so far the diffusion model could only be applied to computerized tasks that allow assessing accuracy and RT for single trials. Given that paper-based tests are still commonly used in cognitive aging research and practice, this study aimed to demonstrate the utility of the diffusion model for paper-based tests of processing speed by applying new technologies (i.e., digital pens) in a lexical decision task.

### 1.4. Research Questions and Hypotheses

Based on the reviewed literature, this study aimed to answer the following research questions:

1. Can age-related differences in processing speed be investigated from a comprehensive diffusion model perspective in a paper-based lexical decision experiment using digital pens?
2. To what degree do results compare to findings previously obtained with the diffusion model in computer-based assessment?

Based on previous work by Ratcliff and colleagues [8] using the lexical decision task, we hypothesized age differences would appear in the accuracy of responses, the RTs, the decision criterion and the nondecision time, but not in the rates of accumulation of evidence (i.e., the efficiency of the decision process).

## 2. Experimental Section

### 2.1. Participants

Twenty younger (50% female) and twenty older (50% female) adults participated in the experiment. The younger adults were university students. They were recruited via the Facebook group of psychology students at the Goethe University, Frankfurt, and mainly participated for course credit in an introductory psychology course. The older adults were healthy, active, community-dwelling seniors who were recruited from the University of the Third Age at the Goethe University, Frankfurt. The older adults and few younger adults who had already obtained their course credits were paid a total of 45 Euro for their participation. Subjects were tested individually in the first session and in a group of a maximum of six participants (either younger or older) in the second session. The majority of participants (35 out of 40) were native German speakers. Five participants spoke other European languages as their mother tongues (e.g., Russian, Polish). These five participants had all learned German during school and spoke German fluently. The means and standard deviations for standard background characteristics are presented in Table 1.

### 2.2. Procedure

**Figure A1:**
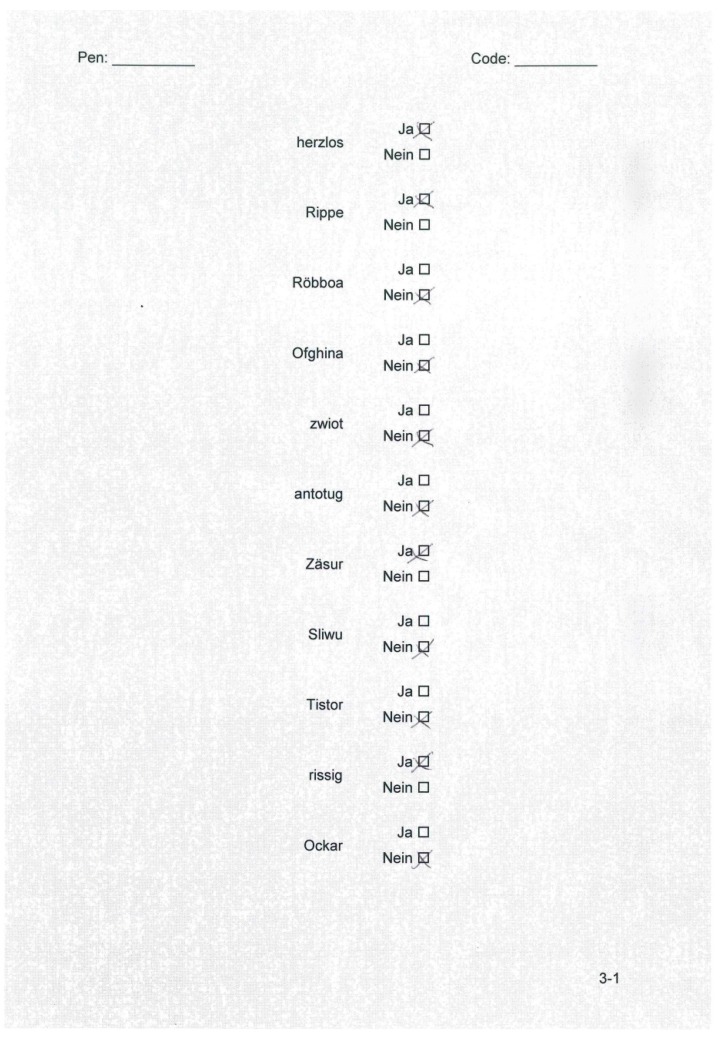
Illustration of one page of the lexical decision task.

The experiment was spread across two parts. In the first part, lasting about one hour, background characteristics were assessed via paper-and-pencil questionnaires and tests. In the second part, lasting about 1.5 to 2 h, the lexical decision task was administered on paper using digital pens. In this task participants were asked to evaluate whether a series of letters represented a German word. Participants answered by checking either “yes” or “no” after each stimulus. They were instructed that the correct spelling, including capitalization, mattered for their decision and that they should answer as fast and as accurate as possible. A total of 67 sessions including 33 stimuli were presented, resulting in a total of 2211 stimuli. Each session contained three pages with 11 stimuli per page printed from top to bottom. The stimuli were presented on the left side of the page and the checkboxes for answering yes or no were positioned to the right of each stimulus. Participants looked at the stimuli and were instructed to work on the stimuli in order of presentation, starting from the top of the page. The stimuli were typed in Arial with a font size of 12. An example of one page of the task is presented in the appendix (see Figure A1). The first stimulus on each page was excluded from the analyses, since it served as a reference for the response times on that page. Excluding the reference stimuli, each session contained 15 words and 15 nonwords that were chosen from the pool of words and nonwords balancing the number of letters. The 201 reference stimuli were chosen randomly from the total number of word and nonword stimuli. Excluding the reference stimuli, 2010 stimuli remained that were treated in the analyses. The first two sessions served as testing sessions and were not included in the analyses. Participants were instructed to answer as fast and as accurately as possible, as well as to take breaks between the sessions by putting the pen down and relaxing their hands. All sessions were conducted in the laboratory by trained research assistants. The study was approved by the local ethical review board.

### 2.3. Stimuli

The stimuli were 1007 low- and very low-frequency words and 1003 nonwords. There were 46.7% low-frequency words with frequencies from more than 1 to 5 per million (CELEX, Celex lexical database, [29]) and 53.3% very low-frequency words with frequencies of 1 per million. All words were selected from CELEX using the software WinWordGen 1.0 [30] and pretested in a pilot study with 11 younger and older participants who had at least secondary school education (i.e., German Abitur). Only those words that were known by all participants were retained for this study. Besides word frequency, the word length was controlled in that it varied between five and seven letters. Given that a large pool of rare words was needed, no further constraints were put on the word selection process. From each word, a nonword was generated by randomly replacing all vowels with other vowels (except u after q and replacing German umlauts by single vowels).

### 2.4. Apparatus

**Figure 2:**
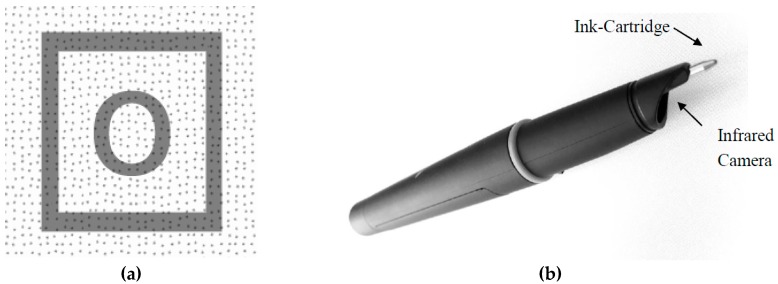
The dot pattern behind this magnified printed rectangle and letter “o” (**a**) is used by the infrared camera built in Anoto Digital Pens (**b**) to uniquely identify its location.

Data collection was conducted using Anoto Digital Pens [24]. Stimuli were presented on regular paper printed on top of a special Anoto dot pattern (see Figure 2a). Digital pens (see Figure 2b) were used to collect responses as well as to process data from the paper-based assessment based on all coordinates and time stamps of strokes drawn on the paper. The digital pens were used in the same way as usual pens, leaving visual traces on the paper, while wirelessly connected to a computer which was used as a receiver. Item level response times were computed from time differences between different strokes that belonged to a particular response. These computed response times for each stimulus were applied to diffusion model analyses.

### 2.5. Background Measures

In the first session, a demographic questionnaire was presented assessing age, sex, and level of education of the participants. Moreover, processing speed was assessed using the digit-symbol substitution coding test [31]. Vocabulary was tested with a German version of the Mill Hill Vocabulary Scale [32]. As has been typically reported in cognitive aging research [33,34], older adults showed worse performance than younger adults in a test of processing speed [i.e., digit-symbol substitution coding, 24; *t*(38) = 4.95, *p* < 0.05], but outperformed younger adults in a vocabulary test (30; *t*(38) = −3.28, *p* < 0.05), see Table 1.

### 2.6. Estimation of the Diffusion Model Parameters

The diffusion model was fit to the experimental data by applying a multidimensional search algorithm in which the fit between predicted and empirical RT distributions (correct and incorrect) was optimized. The parameter search was done by applying a SIMPLEX routine and quantifying the fit following the Kolmogorov-Smirnov (KS) algorithm by use of the software fast-dm [16]. The following parameters of the diffusion model were estimated: the relative starting point of the diffusion process (*z_r_*), the boundary separation (*a*), the nondecision time (*t*_0_), the drift rate (*v*), the across-trial variability in starting point (*s_zr_*), the across-trial variability in nondecision time (*s_t_*), and the across-trial variability in drift rate (*s_v_*). Whereas the drift rate and the relative starting point were estimated separately for the word and the nonword conditions, all other parameters were held constant across stimulus conditions. Thus, drift rate could be affected by the stimulus quality. The relative starting point was allowed to vary across conditions, as this considerably improved model fit (see below). The within-trial variance of drift rate was set to *s* = 1 which is the default in fast-dm [32]. The parameters were estimated separately for each individual RT distribution, avoiding the often applied averaging of RTs across subjects (see [8]).

### 2.7. Data Analysis

In the data analyses, RTs smaller than 250 ms and greater than 3500 ms were eliminated based on careful consideration of the RT distributions. Applying these criteria, 2% of the data were eliminated. Mixed-effects analyses of variance with age group (younger vs. older adults) and condition (words vs. nonwords) were conducted for correct RTs and the accuracy of responses, as well as for all diffusion model parameters that were not constrained across conditions (i.e., drift rate and the relative starting point). Significant interactions were followed up by pairwise comparisons adjusted to avoid family-wise alpha error inflation using Bonferroni corrections. Age differences in boundary separation, nondecision time, and the inter-trial variability parameters, which were held constant across conditions, were analyzed using *t*-tests. Unless otherwise indicated, the level of significance was *p* < 0.05.

## 3. Results and Discussion

### 3.1. Results on RT and Accuracy

A summary of the results concerning RTs and accuracy of responses is presented in Table 2. Descriptively, older adults were slower and their responses to word stimuli were more accurate than those of younger adults. Considering the response times, the older participants were somewhat slower than the younger participants: their correct RTs to words were on average 14 ms longer and their correct RTs to nonwords on average 68 ms. Considering the accuracy of responding, older adults made fewer errors in responding to words than younger adults, whereas there was only a small age difference in accuracy for nonwords.

Analysis of the correct RT data yielded no significant main effect of age group, *F*(1, 38) = 0.64, but a significant effect of stimulus condition, *F*(1, 38) = 21.10, η^2^ = 0.33. Further, there was a reliable Age × Condition interaction, *F*(1, 38) = 5.23, η^2^ = 0.08. Pairwise comparisons revealed that older adults responded significantly slower to nonwords than to words, *t*(38) = 4.87, η^2^ *=* 0.38 (*M* difference = 82.35). All other simple effects were non-significant. Thus, these results showed that despite descriptive age-related differences, older adults were not significantly slower than younger adults in their correct responses to word and nonword stimuli. Moreover, older adults took longer for their correct answers to nonwords than to words.

Analysis of the accuracy of responses yielded a significant main effect of age group, *F*(1, 38) = 5.84, η^2^ = 0.13, but no significant effect of stimulus condition, *F*(1, 38) = 3.07. Further, there was a reliable Age × Condition interaction, *F*(1, 38) = 13.93, η^2^ = 0.25. Pairwise comparisons revealed that older adults were significantly more accurate in their responses to words than younger adults, *t*(38) = 4.13, η^2^ *=* 0.31 (*M* difference = 0.07). Moreover, younger adults responded significantly less accurately to words than to nonwords, *t*(38) = 3.88, η^2^ *=* 0.28 (*M* difference = 0.06). Thus, these results indicated that older adults were more accurate in identifying word stimuli than younger adults but the groups did not differ significantly in identifying nonwords. Besides these age-related differences, younger adults better identified words than nonwords.

### 3.2. Results from Diffusion Model Analyses

A summary of the results concerning diffusion model parameters is presented in Table 3. Analysis of the diffusion model revealed that the older adults differed in their responding from younger adults in several ways. Firstly, the estimate of the nondecision time parameter was larger for older than for younger adults by 53 ms, *t*(38) = −2.35, η^2^ = 0.13. Secondly, drift rates were higher for older adults than for younger adults, *F*(1, 38) = 6.37, η^2^ = 0.14, and also higher for nonwords than for words, *F*(1, 38) = 490.06, η^2^ = 0.92 (note that drift rates for nonwords have the opposite sign from drift rates for words. For the purpose of the ANOVAs, only the absolute differences between the drift rates matter because better performance is indicated by more positive drift values for words and more negative drift values for nonwords. Thus, we converted the drift rates of nonwords to have the same sign as the drift rates for words). These main effects were qualified by a significant Age × Condition interaction, *F*(1, 38) = 6.37, η^2^ = 0.12. Pairwise comparisons revealed that the age-related difference in drift rates was significant for words, *t*(38) = −2.78, η^2^ = 0.17 (*M* difference = 0.56), but not for nonwords (*M* difference = 0.13). Besides the age-related differences, younger adults had significantly larger drift rates for nonwords than for words, *t*(38) = 3.87, η^2^ = 0.28 (*M* difference = 0.47). Thirdly, boundary separation was greater for older than for younger adults, *t*(38) = −3.66, η^2^ = 0.26 (*M* difference = 0.59). Fourthly, the relative starting point differed between stimulus conditions *F*(1, 38) = 151.86, η^2^ = 0.79. Pairwise comparisons revealed that the relative starting point was greater for words than for nonwords for both younger adults, *t*(38) = 7.58, η^2^ = 0.61 (*M* difference = 0.36), and older adults, *t*(38) = 9.71, η^2^ = 0.71 (*M* difference = 0.47). Fifthly, the variation in drift rate across trials did not differ between younger and older adults, *t*(38) = −1.75, η^2^ = 0.07, indicating that there were no age-related differences related to variation of attention or practice effects across trials. Finally, there were also no age-related differences in inter-trial variability of nondecision time, *t*(38) = 0.61, η^2^ = 0.01, and relative starting point, *t*(38) = 1.70, η^2^ = 0.07. Taken together, the main differences between younger and older adults were the slightly longer non-decision component of processing, the better accumulation of evidence as indicated by larger drift rates in the word condition, and more conservative responding for the older adults. Concerning stimulus effects, another main result was that the relative starting points were greater for words than for nonwords for both age groups. This parameter was estimated separately for the two stimulus conditions because this improved model fit for a considerable number of participants (see below). As we had no hypothesis regarding these potential differences in relative starting point for words versus nonwords, the interpretation of this effect can only be speculative. In our task setup, participants could potentially look ahead to the next stimulus, while still executing the response of the previous trial. Providing the sensory encoding with such a head start can be speculated to lead to information accumulation towards a word or nonword response before the controlled decision process starts. The finding that the biases in relative starting point were quite symmetric around the midpoint of 0.5 and towards the correct response speaks for such an interpretation.

### 3.3. Fits of the Diffusion Model to the Data

We assessed goodness-of-fit for a total of 40 models (i.e., one model for each participant) using KS-tests [35]. A model’s fit was indicated by non-significant outcomes at the *p* < 0.01 level (i.e., significant *p*-values indicating misfit). Applying this test, out of the 40 models estimated, 8 had *p*-values < 0.01. However, as discussed by Voss and colleagues [17], the results of the KS-test depend on the number of trials and large datasets (i.e., experiments with many trials) will almost always overestimate model misfit. Thus, we simulated 1000 random datasets based on the 40 datasets (utilizing the mean and covariance of model parameters and number of trials) and fit each dataset to a model based on the empirical model outlined above. The critical fit value was determined to be *p*_fit_ = 0.0227 and based on this revised value ten models were identified as poor fit. Excluding these ten cases from the analyses led to largely similar results with few exceptions. Firstly, for correct RTs the Age × Stimulus interaction failed to reach significance, *F*(1, 28) = 2.79. Secondly, concerning the accuracy of responses, the main effect of Stimulus condition reached significance, *F*(1, 28) = 5.32, η^2^ = 0.13. Thirdly, concerning the drift rate parameter, the main effect of Age group was no longer statistically significant, *F*(1, 28) = 1.06, the Age × Stimulus interaction failed to reach significance, *F*(1, 28) = 3.79, and, as a consequence, pairwise comparisons following up the Age × Stimulus interaction did not find an age-related difference in drift rate for words, *t*(28) = 1.74.

**Figure A2:**
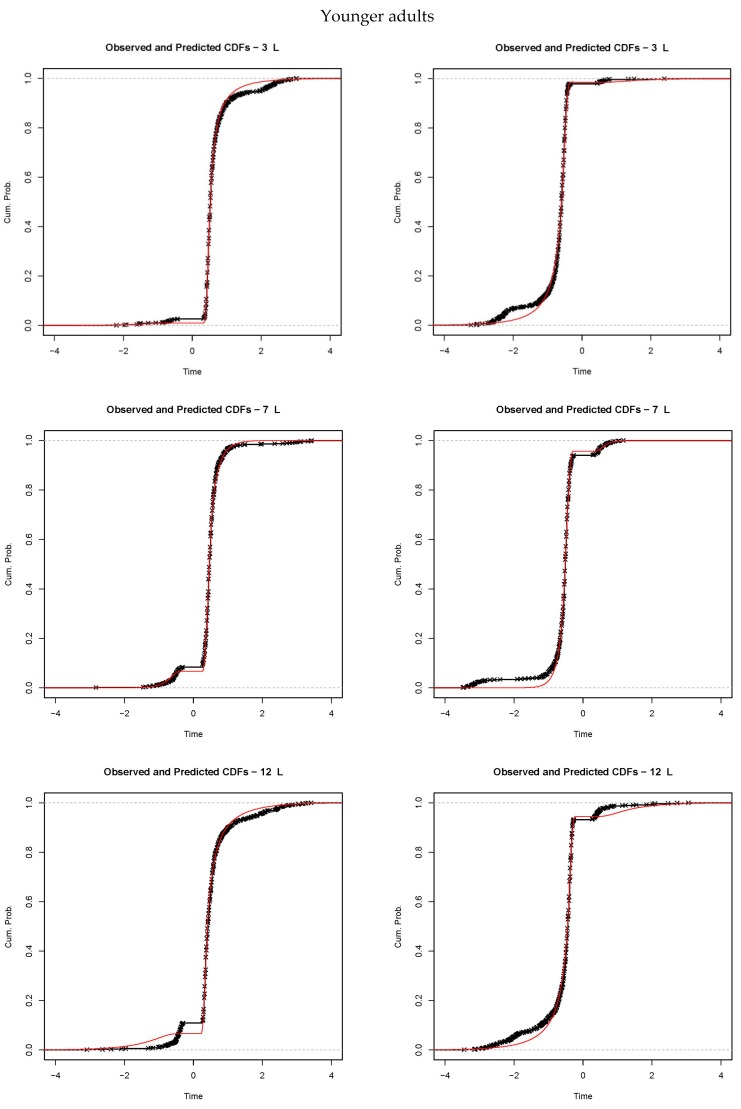

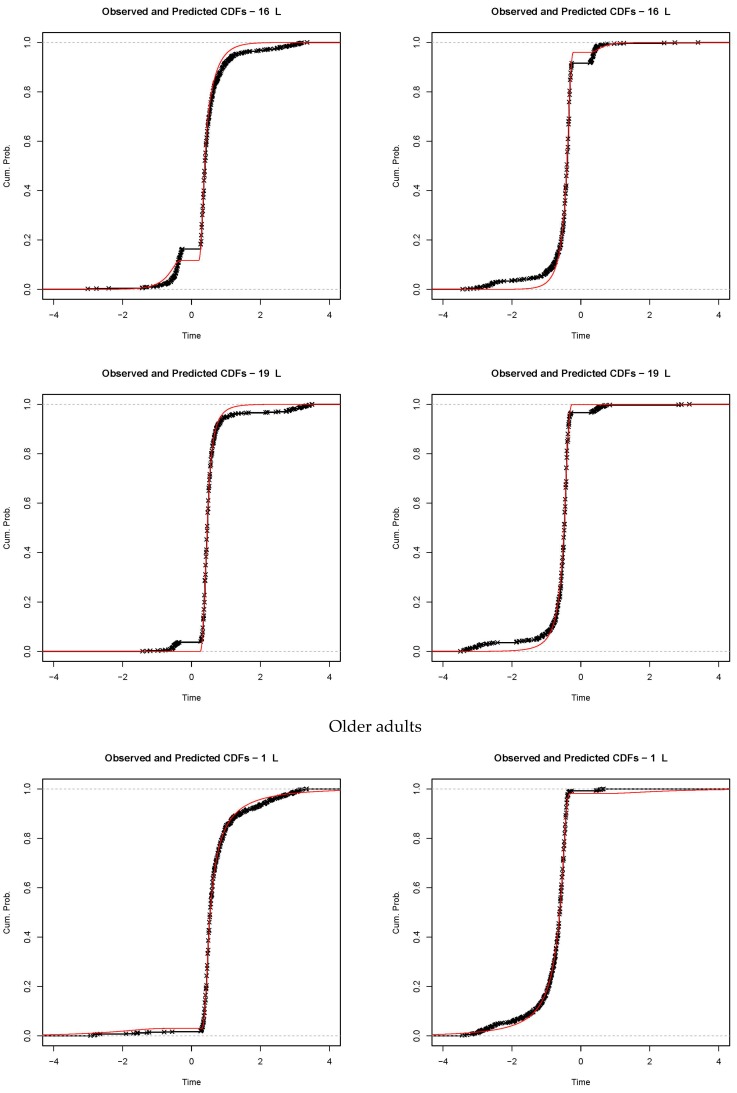

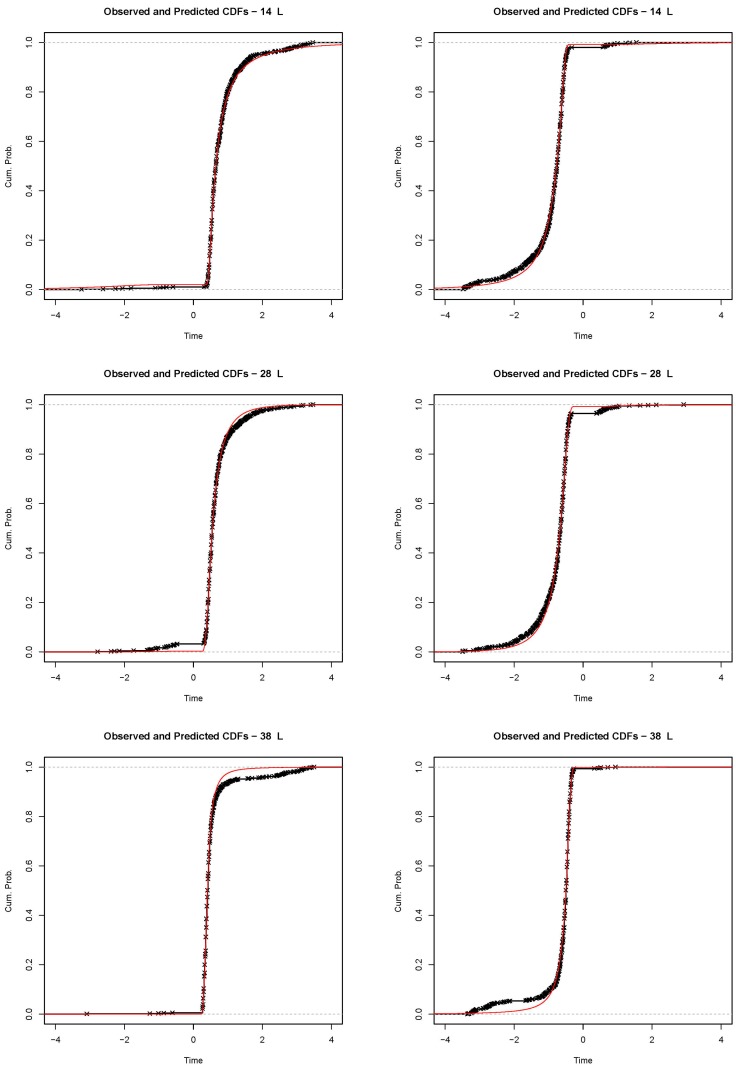

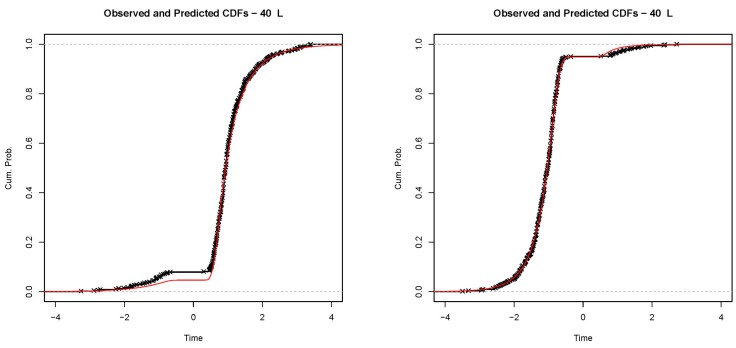
Comparison of the empirical and the predicted cumulative density function (CDF) for words (**left**) and nonwords (**right**). Note that the red line represents the predicted CDF and the crossed line represents the observed CDF.

In addition, we also addressed the question of model fit by graphical inspection. Figure A2 shows the empirical and predicted cumulative density functions for the word condition (left) and the nonword condition (right) for five younger and five older participants that were randomly chosen from the overall sample. Besides some deviations between empirical and predicted densitiy curves for very fast and very slow RTs, the overall fits seem acceptable for both word and nonword conditions for the majority of cases.

As a further control, we checked results for a model with only one relative starting point, despite a larger number of poorly fitting participants. Results were similar, but the age-related differences in boundary separation and nondecision time were not statistically significant.

### 3.4. Post-Hoc Analyses

**Figure 3:**
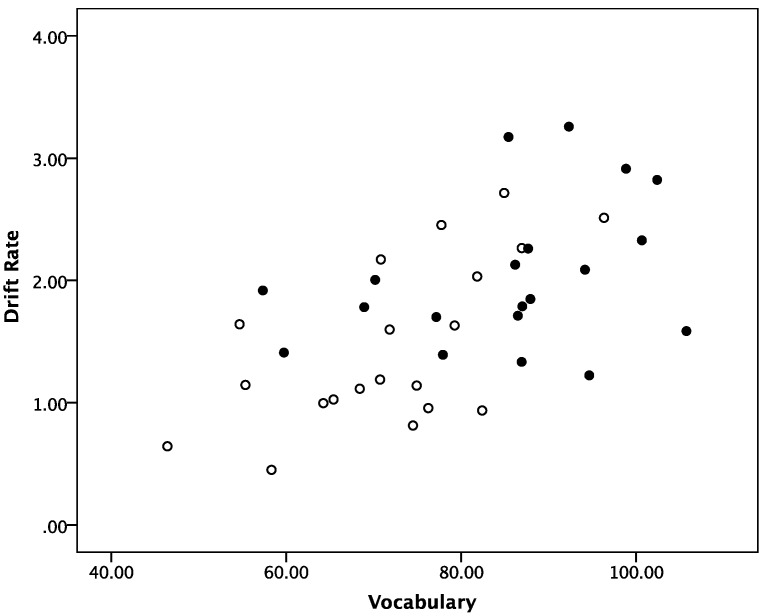
Relationship between drift rate and vocabulary by age group. Note: open circles = younger adults; filled circles = older adults.

Given that older adults demonstrated clear advantages in responding to words, post-hoc analyses were conducted to reveal whether individual differences in vocabulary might elucidate this effect. Bivariate correlations showed that the drift rate for words was significantly positively related to vocabulary, *r* = 0.59 in the overall sample, confirming that individuals with better vocabulary also demonstrated a higher accumulation of evidence for word stimuli (note that by excluding poorly fit cases the correlation dropped slightly to *r* = 0.50). Partial correlations, after controlling for age-related variance, yielded that the positive relationship between the efficiency of evidence accumulation and vocabulary holds independent of age group, *r* = 0.49, thus confirming that generally better vocabulary supports the efficiency of processing lexical information (note that by excluding poorly fit cases the partial correlation dropped slightly to *r* = 0.43). The results are illustrated in Figure 3.

### 3.5. Discussion

Taking into account both accuracy and RT information of correct and error responses, we applied the diffusion model to extract values of the parameters for the different components of processing involved in a lexical decision. The results showed, first, that younger and older adults did not differ in their response times; second, that the older adults applied more conservative decision criteria; third, that older adults did not differ from younger adults in the accumulation of evidence; and fourth, that the nondecision component of RT was on average 53 ms longer for the older than for the younger adults.

These findings partly replicate results previously reported by Ratcliff and colleagues [8] in a computer-based lexical decision experiment. Overall, these age-related differences are in the same direction as those reported by Ratcliff and colleagues, but differ importantly in size and statistical significance. As reported by Ratcliff and colleagues [8], for lexical decision but also for many other experimental paradigms (e.g., signal detection: [36]; letter discrimination: [37]), older adults adopted more conservative decision criteria. This more conservative way of responding might reflect older adults’ higher levels of conscientiousness [38] increasing the emphasis on accuracy of responses. Surprisingly, there were only small age-related differences in response times.

Older adults showed better accumulation of information for words than younger adults. However, this difference in drift rates was no longer statistically significant when considering only those cases which showed good model fit. Ratcliff and colleagues [8] had found an age-related difference in the accumulation of evidence for words and nonwords, with drift rates of older adults being 4.7% larger than those of younger adults; but this difference also did not reach statistical significance.

Nondecision times were numerically somewhat larger for the older adults, with a difference of about half the size reported by Ratcliff and colleagues [8] for their computer-based experiments (53 ms vs. 80–100 ms). Given that nondecision time combines aspects of sensory encoding and motor execution that are related to the modes of presentation (computer screen versus paper) and responding (computer keyboard versus pen marks), this parameter is likely to show different results for the different modes. Age group differences in this parameter are difficult to interpret, however, as potential age-related differences in the sensory encoding of computer- versus paper-based stimuli and the motor execution of keystrokes versus pen marks are convoluted. A possible interpretation for the slightly different numbers is that age-related differences in the experience with paper-based testing are smaller than in the experience with computer-based testing. However, as our experiment lacks a direct comparison of the two modes for the same participants, such interpretations can only be tentative.

Findings also showed differences in starting point biases between word and nonword conditions. Differently from previous modeling approaches with the diffusion model, we let the starting point vary between stimulus conditions to allow for potential bias towards answering yes or no depending on whether participants prejudged the forthcoming stimulus to be a word or a nonword. This was possible due to the presentation of 11 stimuli per page in the paper-based version of the lexical decision task administered in this study. As participants could already see the next stimulus, while still executing the response of the previous trial, sensory encoding of the stimulus could already start. If this head start only affected sensory encoding, however, it could be accounted for by a shortening of the nondecision time component. Finding that separately estimating relative starting point for words and nonwords improved model fit for a substantial number of participants suggests that decision-related information is accumulated before the controlled decision process starts (when the response execution of the previous trial is finished). We are aware that this is a rather speculative account, which calls for deeper investigation in additional experiments, and that, alternatively, the difference in relative starting point might also stem from unidentified sources of misfit that bias relative starting point via parameter dependencies.

Overall, the different modes of presentation (i.e., paper-based in this study vs. computer-based in Ratcliff and colleagues’ work) might explain differences in the findings. Future studies should address this potential mode effect by applying a repeated measures design in which the same participants are assessed twice with the same experimental paradigm, once on paper and once at the computer.

## 4. Conclusions

This experiment demonstrates the possibility to fit theoretical models of cognitive processes in paper-based tests using technology-based assessments to collect process data. Previously found results from computer-based experiments could be partly replicated. Generally, digital pens are a promising data collection tool, reducing differences in the analytical potential between computer- and paper-based experiments. In particular, the technology opens the door to response time analyses at item level. Future studies should address differences between the two modes (computer vs. digital pen) rigorously by implementing an experimental mode effect design. This will also shed some light on whether previous findings based on diffusion modelling (e.g., age differences) depend on the mode of the experiment. Moreover, the generalizability of the reported findings should be addressed in larger representative samples. Finally, model fit should be further evaluated (e.g., by graphical inspection, [17]). The poor fit of some of the models might be due to the nature of the task: due to the paper-based assessment, drift rates might be more variable than typically reported from diffusion model analyses in which response times are based on keyed responses from computer-based assessment. In sum, the applied technology-based assessment using digital pens offers new possibilities to assess age-related differences in processing speed across the lifespan, applying the diffusion model to experimental paradigms that can be implemented on paper (e.g., digit symbol coding). However, it is important to note that many two-choice tasks to which the diffusion model has been applied (e.g., signal detection, brightness discrimination) cannot be realized in paper-based assessment.

## Figures and Tables

**Table 1:** Subject characteristics.

Measure	Younger Adults				Older Adults			
	*M*	*SD*	*Min*	*Max*	*M*	*SD*	*Min*	*Max*
Age	25.70	4.78	18.00	36.00	68.10	3.14	64.00	75.00
Years of education	13.00	1.92	10.00	17.00	14.90	3.58	8.00	20.00
Vocabulary	72.06	12.23	46.42	96.32	85.37	13.41	57.33	105.74
Processing speed	66.05	11.01	49.00	82.00	50.10	9.43	35.00	68.00

**Table 2:** Summary of reaction times (RT) and accuracy data.

Group and Condition	Accuracy	*M* RT Correct (*SD*)	*M* Error RT (*SD*)	*M* Observations (*SD*)
1. Younger adults				
Words	0.91 (0.07)	709 (184)	1030 (359)	985 (16)
Nonwords	0.98 (0.02)	737 (188)	994 (457)	989 (11)
2. Older adults				
Words	0.98 (0.02)	723 (138)	1227 (314)	938 (119)
Nonwords	0.96 (0.07)	805 (149)	1024 (234)	954 (99)

**Table 3:** Summary of diffusion model parameters.

Group and Condition	*v*	*a*	*t*_0_	*z_r_*	*s_v_*	*s_t_*_0_	*s_zr_*
Younger adults (words)	1.472 (0.675)	2.106 (0.489)	0.345 (0.073)	0.724 (0.089)	0.662 (0.191)	0.183 (0.052)	0.256 (0.088)
Younger adults (nonwords)	−1.944 (0.645)			0.359 (0.123)			
Older adults (words)	2.033 (0.602)	2.679 (0.540)	0.399 (0.071)	0.771 (0.107)	0.758 (0.153)	0.172 (0.062)	0.215 (0.062)
Older adults (nonwords)	−2.069 (0.545)			0.305 (0.125)			

*Note:* Mean values of the parameter estimates are presented. Values in parentheses refer to the standard deviations of the mean parameter estimates.
